# Oral SARS-CoV-2 Spike Protein Recombinant Yeast Candidate Prompts Specific Antibody and Gut Microbiota Reconstruction in Mice

**DOI:** 10.3389/fmicb.2022.792532

**Published:** 2022-04-07

**Authors:** Lilin Zhang, Lan Yao, Yanyu Guo, Xiaoyang Li, Li Ma, Ruiqi Sun, Xueqing Han, Jing Liu, Jinhai Huang

**Affiliations:** ^1^School of Life Sciences, Tianjin University, Tianjin, China; ^2^Tianjin Institute of Pharmaceutical Research Co., Ltd., Tianjin, China; ^3^Chinese Academy of Inspection and Quarantine, Beijing, China

**Keywords:** *Saccharomyces cerevisiae*, SARS-CoV-2, spike protein, oral administration, microbiome reconstruction

## Abstract

A recent study showed that patients with coronavirus disease 2019 (COVID-19) have gastrointestinal symptoms and intestinal flora dysbiosis. Yeast probiotics shape the gut microbiome and improve immune homeostasis. In this study, an oral candidate of yeast-derived spike protein receptor-binding domain (RBD) and fusion peptide displayed on the surface of the yeast cell wall was generated. The toxicity and immune efficacy of oral administration were further performed in Institute of Cancer Research (ICR) mice. No significant difference in body weights, viscera index, and other side effects were detected in the oral-treated group. The detectable RBD-specific immunoglobulin G (IgG) and immunoglobulin A (IgA) of severe acute respiratory syndrome coronavirus 2 (SARS-CoV-2) and more complex microbiota were detected from oral administration mice compared with those of the control group. Interestingly, the recombinant yeast was identified in female fetal of the high-dose group. These results revealed that the displaying yeast could fulfill the agent-driven immunoregulation and gut microbiome reconstitution. The findings will shed light on new dimensions against SARS-CoV-2 infection with the synergistic oral agents as promising non-invasive immunization and restoring gut flora.

## Introduction

The global coronavirus disease 2019 (COVID-19) outbreak and the emergence of multiple severe acute respiratory syndrome coronavirus 2 (SARS-CoV-2) variants have seriously threatened public health and socioeconomic stability. An intensive effort has been underway to develop countermeasures, including vaccines, neutralizing antibodies, and antiviral drugs (Su et al., 2020).

Vaccination is one of the most cost-effective and successful public health interventions to prevent infections and save millions of lives (Orenstein and Ahmed, 2017). Currently, three main types of COVID-19 vaccines are authorized and recommended or undergoing large-scale (Phase 3) clinical trials.^1^ Most of them are designed to be delivered by the parenteral route to produce high titers of systemic neutralizing antibodies to cope with the systemic viral infection (Folegatti et al., 2020; Gao et al., 2020; Logunov et al., 2020; Wang et al., 2020; Zhu et al., 2020). However, this strategy leaves some questions about the durability and efficacy of the mucosal immune response after vaccination which is essential for blocking viral entry through oro-respiratory tracts (Ashraf et al., 2021). Oral vaccines have been well established for preventative immunity against several respiratory infectious diseases, not only inducing mucosal secretory immunoglobulin A (sIgA) antibody and serum antibody [immunoglobulin G (IgG)] responses but also delivery for intestinal protective immunity against infections at rectal and genital mucosae (Zhu and Berzofsky, 2013; Kang et al., 2018).

The gut is the largest immunological organ in the body, and its resident microbes are known to influence immune responses. Patients with COVID-19 have been reported to have gastrointestinal symptoms and intestinal flora dysbiosis during hospitalization and up to 30 days after recovery, which indicated that gut microbiota could have important implications for long COVID-19 (The, 2021; Yeoh et al., 2021). Human gut-lung microbiota interplay provides a framework for treating and managing infection and posts COVID-19 syndrome. The regulation of intestinal flora may be helpful for recovery from sickness. The efficacy of the microbiome immunity formula in improving immune function is under clinical trials recruiting (NCT04540406, NCT04884776).

Brewer’s yeast has a generally recognized as safe (GRAS) status with the FDA. It can function as a probiotic in modulating gut microbiota (Xu et al., 2018), also inducing mucosal immunity based on notably β-glucans (BGs) and mannan on its cell wall. In our previous study, two kinds of yeast chassis designs for exhibiting ASFV (Chen et al., 2021) and PCV2 (Chen et al., 2018) oral vaccine candidates, efficacy preventing African swine fever virus infection, and porcine circovirus type 2 poses.

In this study, the spike protein-containing receptor-binding domain (RBD) and fusion peptide (FP) domain of SARS-CoV-2 were exhibited on the surface of a yeast cell with an Aga1-Aga2 anchor. Also, we interrogated the immunogenicity and the influence of intestinal flora by orally delivering the yeast candidate to mice. Results showed that yeast agents induced both mucosal antibody response immunoglobulin A (IgA) and systemic antibody responses (IgG) and altered gut microbiome compositions after administration. Taken together, our data highlight the possibility of oral yeast agents as promising non-invasive immunization candidates used in cocktail vaccines responses to SARS-CoV-2 infection.

## Materials and Methods

### Plasmid Construction

**FIGURE 1:**
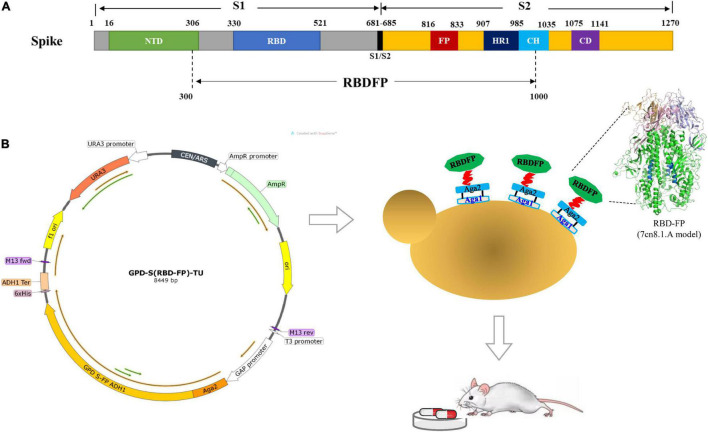
Construction of spike S [receptor-binding domain (RBD)-fusion peptide (FP)] expression vectors. **(A)** Schematic diagram of severe acute respiratory syndrome coronavirus 2 (SARS-CoV-2) spike protein expression construct. **(B)** Diagram of the chromosomal region, including the S (RBD-FP) transcriptional unit with *GAP* promoter and *ADH1* terminator fused to the C-terminal of Aga2 for presentation on the yeast cell surface. The structure of RBD-FP subunit was predicted by SWISS-MODEL based on PDB 7cn8 as the template.

The GPD-S (RBD-FP)-TU recombinant plasmid contains 300–1,000 amino acid residues of the SARS-CoV-2 spike protein (GenBank: MT407658.1), including RBD (330–521 aa) and viral FP (816–833 aa). It contains a hexahistidine tag at the C-terminus. The S (RBD-FP) DNA fragment was synthesized (GENEWIZ, Beijing, China) and ligated with a pET28a plasmid. Then, S (RBD-FP) DNA sequence was sub-cloned into the yeast expression vector pGPD-ADH1-POT (Chen et al., 2021) by overlapping PCR with specific primer pairs harboring homologous sequences in the vector (CoV-S Forward: gacgataaggtaccaggATCCATGAAGTGTACGTTGAAATCCT; CoV-S Reverse: gaattccaccacactggatccTCTGCCTGTGATCAAC CTAT) (Figure 1). The constructed GPD-S (RBD-FP)-TU plasmid was directly transformed into DH5α competent cells and selected on LB-ampicillin medium. Then, the transcription unit of S (RBD-FP) was released with *Bsa*I, leaving overhangs compatible with URR1, URR2, and LEU parts (Guo et al., 2015). The recombinant cassette was transformed into *Saccharomyces cerevisiae* ST1814G using the LiAc/ss carrier DNA/PEG method as described (Gietz and Woods, 2002). Transformants were selected on synthetic-defined (SD) medium minus Leu plates and incubated at 30°C for 72 h. Six independent clones of ST1814G-S (RBD-FP) were tested.

The genotyping assay was performed by genomic DNA extraction (Looke et al., 2011) from recombinants and PCR using primer pair (S-F 331: AATATTACAAACTTGTGCCCT and S-R 524: AACAGTTGCTGGTGCATGTAG). Also, the phenotype assay was identified by Western blotting, flow cytometry, and immunofluorescence assay.

### Western Blotting

To evaluate the expression of S (RBD-FP) proteins in the recombinant, yeast cells were cultured in yeast extract-peptone-dextrose (YPD) liquid medium at 30°C for 48 h. The 2-ml culture was centrifuged at 5,000 × *g* for 1 min, then, the pellet in 100 μl lysis buffer (50 mM Tris–HCl [pH 8.0], 2 mM EDTA, 1 mM phenylmethylsulfonyl fluoride) was resuspended, and glass beads were added to break cells five times using the disruptor genie (Scientific Industries, D48-1040). The resulting proteins were separated by sodium dodecyl sulfate-polyacrylamide gel electrophoresis (SDS–PAGE) and electroblotted onto a polyvinylidene difluoride (PVDF) membrane. Then, proteins-enriched membranes were blocked with Tris-buffered saline [TBS with 0.1% Tween-20 (TBST)], added 5% (w/v) non-fat dry milk buffer for 1 h, and incubated with His-specific antibody (Cat. HT501, Transgene, Beijing, China) or spike protein S1-specific antibody (Cat. A20136, ABclonal, Wuhan, China) as the primary antibody (1:5,000 dilution) overnight at 4°C, followed by incubation with Horseradish peroxidase (HRP)-conjugated secondary antibody (Goat anti-Rabbit, Cat. PI31460, Goat anti-Mouse, Cat PI31430, Invitrogen™ from Thermo Fisher Scientific, Carlsbad, CA, United States) for 1 h at room temperature (RT). The PVDF membrane was washed at least three times with TBST. Bound antibodies were detected using the Pierce ECL Western blotting substrate (Cat. 32109, Thermo Fisher Scientific, Carlsbad, CA, United States), and band densitometry was performed with Image Lab software (Bio-Rad, Hercules, CA, United States).

### Immunofluorescence Assay

Yeast transformants were subjected to an indirect immunofluorescence assay with a polyclonal 6*His-Tag antibody (Cat. HT501, Transgene, Beijing, China) and a fluorescein isothiocyanate (FITC)-conjugated goat anti-mouse immunoglobulin as the secondary antibody (Cat. ab6785, abcam, Cambridge, UK). Yeast culture was centrifuged, and the pellet was washed with PBS three times at RT and then incubated with primary antibody (anti-His, 1:5,000 dilution) at RT for 30 min. After the washing step with PBS, the secondary antibody FITC-conjugated anti-mouse IgG (1:500 dilution) was incubated for 30 min. The pellet was washed with PBS and visualized under a laser confocal microscope (UltraView Vox, PerkinElmer). ST1814G served as a negative control.

### Flow Cytometry

The characterization of both the recombinant and the control cells of *S. cerevisiae* strains by flow cytometry has been compiled based on the set of His tag marker as described above. The experimental procedure was similar to the one described in immunofluorescence. Yeast cells were harvested after 48 h and washed three times with PBS. Then, cells were fixed, blocked, and incubated with primary antibody (anti-His, 1:500 dilution) for 1 h at 4°C in a humid chamber. Then, the cells were incubated with secondary antibody conjugated to FITC at RT protected from exposure to light. Fluorescence-activated cell sorting was performed with FACS LSR II (BD Biosciences, United States), and a total of 1.5 × 10^5^ cells were analyzed. The data analysis was available using FlowJo software.

### Yeast Agent Production

A single colony of ST1814G-S (RBD-FP) from freshly streaked SD-His agar was inoculated into 20 ml YPD liquid medium and cultured for 48 h on a rotary shaker at 180 rpm 30°C, while the ST1814G as a negative control. Then, the 20 ml of culture was transferred to incubate a 2-L flask containing 1 L fresh YPD medium, vortexed, and incubated at 30°C for 96 h with shaking.

The culture was centrifuged at 13,000×*g* for 5 min. After removing the supernatant, the freeze-drying pellets were quantified using Western blot and worked as yeast agents for animal assay.

### Animals and Administration

Fifty female and fifty male SPF Institute of Cancer Research (ICR) mice (weight, 19–21 g) were purchased from SPF Biotechnology Co., Ltd. [Beijing, Certificate Number: SCXK (Jing) 2019-0010] and housed at the Tianjin Institute of Pharmaceutical Research Co., Ltd. The animal studies were performed following the recommendations for the care and use of laboratory animals of the national institutes of health. The Institutional Animal Ethical Committee approved the animal protocol of the Tianjin Institute of Pharmaceutical Research [Certificate Number: SYXK (Jin) 2016-0009, Tianjin, China].

### Histopathology and Colonization in Fecal Pellets

Hematological examinations were made on four animals per group at study termination. According to the standard protocol, liver, spleen, kidney, and lung tissue samples were collected for histopathological examination. Procedures were performed using 4% neutral buffered formalin, embedded in paraffin, sequential sectioned to 4-μm thickness, and stained with hematoxylin and eosin (H&E) before examination by light microscopy (Ding et al., 2003). Gross examination and organ weight determinations were made on the same animals.

Colonization was tested over time by collecting fresh fecal pellets from 4 to 24 h after oral gavage and plating homogenates on YPD agar (Pierce et al., 2013). Colony-forming units (CFUs) were measured after 48 h of incubation. The candidate yeast colonies were determined by PCRs with S-F and S-R primers and controlled with Actin-F and Actin-R primers.

### Measurement of Antigen-Specific Immunoglobulin G and Immunoglobulin A Antibodies by Enzyme-Linked Immunosorbent Assay

Spike-specific IgG and IgA titers of polyclonal serum from the immunized mice were measured by Enzyme-Linked Immunosorbent Assay (ELISA), as previously described (Chen et al., 2021). In brief, 96-well ELISA plates were pre-coated with prokaryotic expression RBD protein (0.2 μg/ml) overnight at 4°C. After blocking and incubating with serially dilution mice sera, bound IgG and IgA antibodies were detected using HRP-conjugated anti-mouse IgG (1:10,000). The optical density (OD) at 450 nm was determined using a microplate spectrophotometer. The levels of antigen-specific antibodies were determined by calculating the positive/negative (P/N) value. The significance of the difference in antigen-specific IgG/IgA titers among the groups was an analysis of variance (ANOVA) followed by Tukey’s multiple-comparison test, and a *p*-value of less than 0.05 was examined as statistically significant.

### Gut Microbiome Analysis

Comparative analysis of terminal ileum microbiota between yeast-agent and negative control groups reveals the composition and diversity of the gut microbiota. DNA extraction from terminal ileum combination samples from three mice accorded to the manufacturer’s guidelines and optimized for high-throughput processing. The 16S rRNA gene comprising the V4 region was amplified by PCR using composite-specific bacterial primers (the forward primer 515F: 5′- GTGCCAGCMGCCGCGGTAA-3′ and the reverse primer 806R: 5′-GGACTACHVGGGTWTCTAAT-3′). High-throughput pyrosequencing of the PCR products was performed on an Illumina Nava 6000 platform at Biomarker Technology Co., Ltd. (Beijing, China).

Bioinformatic analysis was performed using BMKCloud^2^ and OmicStudio tools at https://www.omicstudio.cn/tool. High-quality reads for the bioinformatic analysis were performed, and all of the effects reads from each sample were clustered into operational taxonomic units (OTUs) based on a 97% sequence similarity according to USEARCH (Ultra-fast sequence analysis) (Edgar, 2013). OTU-level diversity indices were generated by USEARCH. Non-metric multidimensional scaling (NMDS) is an indirect gradient analysis approach that produces an ordination based on a distance or dissimilarity matrix (Looft et al., 2012). BugBase^3^ was used to calculate differences between both groups in terms of microbial phenotypes, based on high-quality sequences (Ren et al., 2020).

### Statistics

All data are expressed as the arithmetic means and standard deviation (X ± SD). One-way ANOVA (SPSS 17.0) was used for the statistical comparison of the differences in data between the test groups. Values of *p* < 0.05 were considered significant.

## Results

### Cloning and Expression of SARS-CoV-2 Spike (RBD-FP)

The yeast-expressed SARS-CoV-2 spike protein was constructed and exhibited on the surface of yeast cells as previously described (Chen et al., 2018, 2021). The spike protein RBD within the S1 subunit and the FP within the S2 subunit were chosen to offer a potential cross-reactive epitope. Structurally, the extracellular domain of the S1 and S2 subunits forms the bulbous head and stalk region of the spike protein, respectively (Yan et al., 2020). Cryo-EM unveils the FP partially exposed on the surface of the spike trimer both in SARS-CoV and Middle East respiratory syndrome coronavirus (MERS-CoV) (Yuan et al., 2017). Functionally, the FPs are conserved across the viral family, which are inserted into the host cell membrane to trigger the fusion event (Tang et al., 2020). Therefore, the engineering yeast cell containing both RBD and FP domain could be a potential antifusogenics, which would prevent the binding of SARS-CoV-2 S-protein to angiotensin-converting enzyme 2 (ACE2).

**FIGURE 2:**
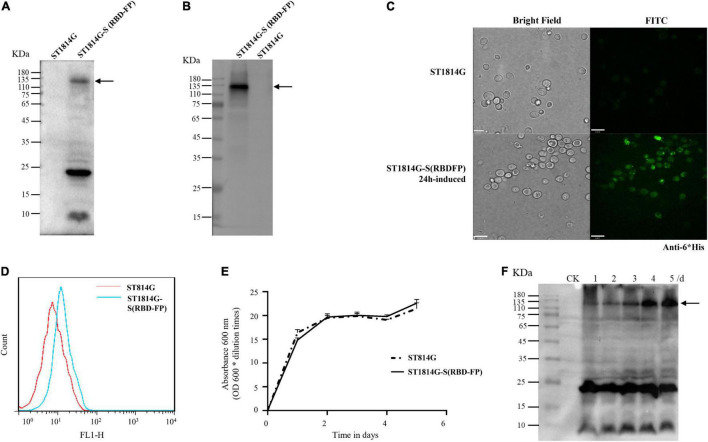
Expression profiles of the spike S (RBD-FP) protein constructed in yeast. The expression of S (RBD-FP) was detected by Western blot with anti-His primary antibody **(A)**, or anti-Spike protein S1-specific antibody **(B)**, immunofluorescence assay **(C)**, and flow cytometry **(D)**. The fermentation kinetics was represented by recombinant yeast concentration measured by spectrophotometric method at the cultivation time points of 1, 2, 3, 4, and 5 days **(E)**. Spike protein expression at each time point was detected by Western blot **(F)**.

To demonstrate if the functional module of GPDp-(Aga2-RBD-FP)-ADH1t works effectively, the genotype and phenotype were determined by PCR, Western blot, immunofluorescence, and flow cytometry (Figure 2). The expressed Aga2-RBD-FP His-tag fusion protein includes 813 amino acids (approximately 89.4 kDa), and the glycosylation sites make the band size appear larger than expected using anti-His tag antibody (Figure 2A) and anti-Spike antibody (Figure 2B) as described (Lo Presti et al., 2020; Zhou et al., 2020; Tian et al., 2021). In addition, fluorescent microscopy (Figure 2C) and flow cytometry (Figure 2D) analysis showed a specific green fluorescence signal in the ST1814G-S (RBD-FP) expression yeast compared with wild-type yeast cells.

### The Fermentation Kinetics of the Recombinant *Saccharomyces cerevisiae* Strain

To maximize the expression yield of the recombinant spike (RBD-FP), the time-course expression profiles of recombinant yeast and its prototype strain were compared to figure out induction time in the YPD medium. Based on the growth curve assay (Figure 2E), there is no significant variation in cell count between the recombinant spike (RBD-FP) strain and the wild-type strain. As shown in Figure 2F, the protein expression of the spike (RBD-FP) increased gradually and reached its peak at 5 days of culture. In detail, the first 2 days were in the logarithmic growth phase with the slightly rising expression protein and then a stationary period of 2–3 days with a dramatically magnified, detectable whole molecular weight of spike protein.

### Yeast Agents Induce Detectable Immunoglobulin A and Immunoglobulin G Responses and Lower the Liver Weight Ratio

**FIGURE 3:**
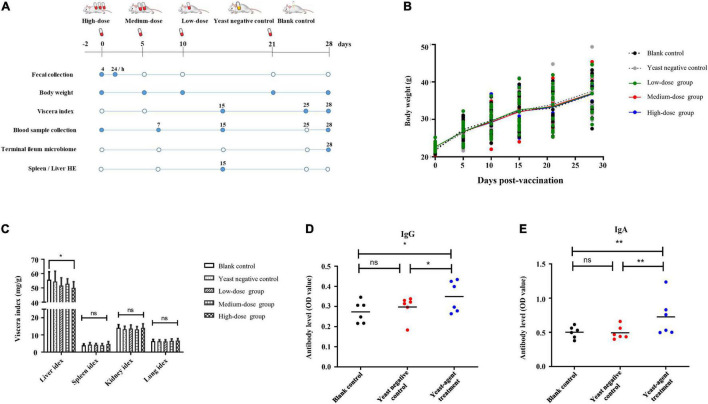
Study overview and effects of yeast agents on body weight, viscera index, and virus-specific antibody responses in vaccinated mice. **(A)** Study overview. A total of 100 mice were randomly divided into five groups and received the yeast agents at 0−, 5−, 10−, 21−, and 28-days intervals *via* oral gavage administration. As illustrated in the diagram, samples were collected at regular intervals (filled circles). **(B)** Body weights were measured at 0−, 5−, 10−, 21−, and 28-days intervals. **(C)** Also, organ weight was measured at day 28, taking 12 mice per group. Viscera index = (Total weight of viscera/total body weight before removing the viscera) * 100. **(D,E)** Vaccinated mice were fed with ST1814G-S (RBD-FP) four times at 5−, 5−, 11−, and 7-days intervals. The titers of spike-specific total immunoglobulin G (IgG) **(D)** and immunoglobulin A (IgA) **(E)** antibodies in serum were evaluated by ELISA. Serum samples were collected at the end of four immunization times. The individual animal response to each antigen was evaluated in triplicate and is depicted as the mean of the absorbance values at 450 nm minus the mean absorbance of cognate pre-immunization serum. The antigen-specific titers among the treatment groups were compared using ANOVA, followed by Tukey’s multiple-comparison test. Error bars show the standard deviation between triplicates. The mean and standard error of antibodies of each group is shown. *P = 0.05 and **P = 0.01.

To investigate the role of the yeast agents in shaping immune response and gut microbiome, 100 mice were randomly divided into five groups (Figure 3A). Freeze-drying yeast pellets were resuspended in sodium chloride injection solution. Feeding studies were performed as follows: (1) high-dose animals were orally administered 15 billion CFU of ST1814G-S (RBD-FP) yeast; (2) medium-dose animals were orally administered 1.5 billion CFU of the recombinant yeasts; (3) low-dose animals were orally administered 0.15 billion CFU of the recombinant yeasts; (4) the same dose as medium-dose of ST1814G was primed as a yeast negative control, and (5) standard diet was treated with blank control animals. Except for the standard diet group, all animals were orally administered on days 5, 10, and 21 for boost immunization. We collected biological samples at regular intervals from vaccinated mice and measured critical aspects of the immune system as described in the “Materials and methods” section and Figure 3A.

**FIGURE 4:**
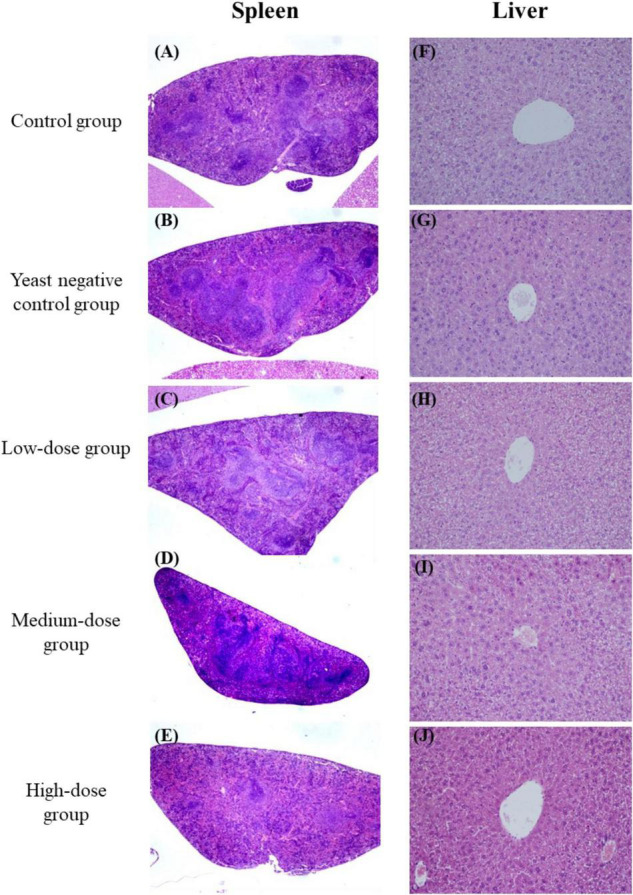
Histopathological examination of the spleen **(A–E)** and liver **(F–J)** from immunized mice. Tissues were collected and stained with hematoxylin and eosin (H&E; original magnification 10×).

There was no difference in body weight between the control groups and all the treated groups after yeast agent administration (Figure 3B). Interestingly, the liver/body weight ratio indicated a statistically significant difference (*p* < 0.05) between the high-dose group and blank control after 25 days of administration (Figure 3C). Yeast exerts a protective function on the liver, also established by Feng’s teamwork, demonstrating that yeast decreases hepatic lipid content and improves the liver function of juvenile largemouth bass (Feng et al., 2022). Furthermore, we also noted that the yeast agents did not induce the infiltration of inflammatory cells, and no specific pathological changes were observed in the liver and spleen of immunized mice (Figure 4). These results reveal that yeast-based vaccines are safe for mice.

This finding promoted the follow-up immunogenicity assessment for the yeast agent. As shown in Figures 3D,E, spike-detectable IgG and IgA antibodies were upregulated as expected after yeast-agents treatment at day 28, compared with blank control and yeast negative control group. These data highlight that mice orally administrated with the yeast agent could induce the specific antibodies throughout the test cycle.

### Significant Alteration of Gut Microbiome Composition After the Yeast Agent Treatment

**FIGURE 5:**
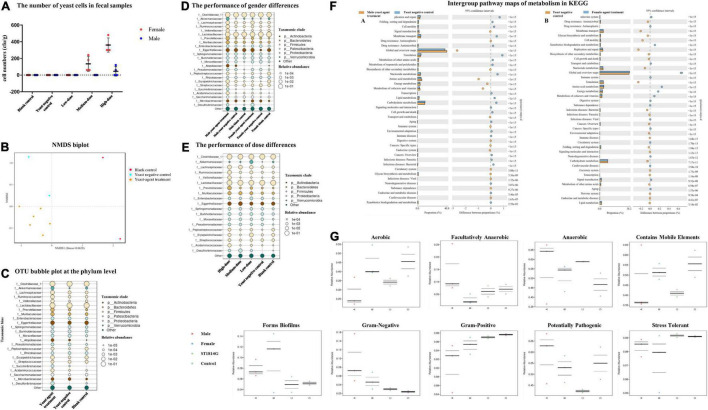
Effects of the yeast agents’ use on the gut microbiome of mice. **(A)** The number of yeast cells in fecal samples. Fecal samples were harvested from 4 to 24 h after oral gavage. Feces were cultured in yeast extract-peptone-dextrose (YPD) liquid by diluting 0.2 g of feces in 1.2 ml YPD liquid at 30°C for 4 h. A 100 μl mixed aliquot was then plated onto YPD agar and repeated three plates. The number of ST1814G-S (RBD-FP) yeast cells was established after 48 h of incubation. **(B)** Non-metric multidimensional scaling (NMDS) biplot of a Bray-Curtis dissimilarity matrix analysis. The yeast agent and blank control groups indicated a significant difference (*P* < 0.05) with the ordination. **(C)** Bubble plots showing the relative abundance of indicator operational taxonomic units (OTUs) in each of the three groups of the sampling stations. The bubble size indicates the relative abundances (%) of each OTU in the sampling station. Consensus taxonomic groups of each indicator OTU are presented on the left side of bubble plots. Bubble plots show the relative abundance of indicator OTUs in gender-based groups **(D)**, and dosage groups **(E)**. **(F)** Intergroup pathway maps of metabolism in KEGG. Comparison between gender in high-dose treatment with ST1814G host cell. **(G)** Intergroup difference proportion at the family level. BugBase determines the ratio of phenotypes in each microbiome sample. The phenotypic types included Gram staining, oxygen requirement, biofilm-forming, potential pathogens, and containing mobile elements.

ICR mice were inoculated with the recombinant yeast by oral gavage to determine the yeast agent’s capability to penetrate the intestinal lining. Fecal pellets were collected from 4 to 24 h post-primary immunization. Interestingly, the high-dose group of female mice accumulated much more recombinant yeast cells in fecal, compared with corresponding male and other groups (Figure 5A). The result indicated that yeast agents could successfully penetrate the intestinal line, mimic a natural infection, and trigger an immune reaction.

Then, we evaluated the effect of oral yeast on gut microbiota. More evidence is emerging that yeast derivatives have a beneficial influence on the immune system and resistance to the colonization of pathogenic microorganisms (Xu et al., 2018; Elghandour et al., 2020). At the same time, gut microbiome composition was significantly altered in patients with COVID-19 than non-COVID-19 individuals (He et al., 2020). To decipher the role of yeast-mediated immunomodulatory potential and determine beneficial gut species, gut microbiome compositions and functional changes were investigated (Figure 5). Raw sequencing data are available in the National Center for Biotechnology Information Sequence Read Archive (NCBI-SRA) repository under the BioProject accession number PRJNA812734.

Samples were sequenced with an average of 76,920 reads per sample. As expected, beta-diversity analysis shows that the microbiota composition was significantly different among the blank control, yeast negative control, and recombinant yeast group (NMDS stress = 0.0628, Figure 5B). The top 25 species at the phylum level belonging to the *Firmicutes*, *Bacteroidetes*, *Actinobacteria*, and *Verrucomicrobia* were more relatively abundant in recombinant yeast-treated groups than the yeast negative control (Figure 5C). In addition, compared with the blank control, yeast negative control groups induced a slight increase in *Proteobacteria* and *Actinobacteria*. We also examined dose-dependent diversity at the phylum level, but no significant difference among the three dosage groups was observed (Figure 5E). Furthermore, gender is also one of the critical variables affecting the gut microbiota. *Succinivibronaceae*, *Atopobiaceae*, and *Akkermansiaceae* dominated the gut microbiota of male-treated subjects, while *Desulfovibrionaceae* were slightly more abundant in female groups (Figure 5D).

### Impact of Yeast Agent on the Metabolism

The gut microbiota plays a vital role in nutrient metabolism, including the digestion of complex carbohydrates and the synthesis of vitamins (Levy et al., 2017). Therefore, the effects of gut microbe on the metabolome were clarified by comparing control and yeast agent groups. As shown in Figure 5F, amino-acid metabolism, energy metabolism, and the metabolism of cofactor and vitamins displayed the top three functions in the administration cohort relative to yeast control subjects as measured by KEGG pathway enrichment analysis. This is consistent with the established procedures of the gut microbiota in regulating the susceptibility to inflammation. The deficiency of ACE2 caused critical impairment of local tryptophan homeostasis in a mouse model and altered tryptophan metabolism into the kynurenine pathway (Thomas et al., 2020), which could alter the intestinal microbiome and susceptibility to inflammation (He et al., 2020). In addition, other kinds of elements, including signal transduction, transport, catabolism, biosynthesis of secondary metabolism, and the endocrine system, are detected in both gender groups. Moreover, xenobiotics biodegradation, immune system, and cancer-related metabolism were unique in the high-dose female group.

Then, we investigated the potential phenotypic differences in all groups, including oxygen requirements, biofilm-forming, Gram stain testing, mobile elements, potentially pathogenic, and stress-tolerant by BugBase (Figure 5G). Among all the phenotypes, recombinant yeast-immunized mice tend to have a significantly higher representation of Gram-negative, related to biofilm-forming, and potentially pathogenic. In addition, male groups contain much more anaerobic and facultative anaerobic bacteria, while females have a wide range of aerobic gut flora.

## Discussion

Due to the rapid spread and body-wide dissemination of the SARS-CoV-2, vaccines and therapy drugs are urgently needed. Over the past few months, many kinds of SARS-CoV-2 vaccine candidates have been under development, and several of them are currently authorized and distributed. Most granted vaccines for COVID-19 are injected intramuscularly or subcutaneously route, and they have a degree of efficacy in humans (70–95%) depending on the type of vaccine (Folegatti et al., 2020; Gao et al., 2020; Logunov et al., 2020; Wang et al., 2020; Zhu et al., 2020). However, less protection is provided against viral replication and shedding in the upper airways due to the lack of sIgA immune response (Krammer, 2020). Nasal vaccination and oral administration with RBD definitely induced a protective immune response in mice (Du et al., 2021; Gao et al., 2021). This study generated recombinant yeast exhibiting RBD-FP by a-agglutinin (Aga1–Aga2) anchor on the cell surface. With the help of yeast characteristics, the oral agent, without adjuvant-induced, elicits an immune response and alternation in gut microbiome composition.

*Saccharomyces cerevisiae* is a well-understood eukaryote, playing an important role in creating foods and developing them into an edible vaccine due to its simple cultural condition and non-toxic nature. The hepatitis B vaccine is the best available evidence (Valenzuela et al., 1982; Kumar and Kumar, 2019). Yeast has been shown to induce protective immunologic responses in mammals and is avidly taken up by dendritic cells (DCs) and macrophages depending on its cell wall components such as BG and mannan, which allows efficient phagocytosis of yeast cells by antigen-presenting cells (APCs) followed by the generation of danger signals during microbial (Olson et al., 1996; Stubbs et al., 2001; Heintel et al., 2003; Noss et al., 2015; Silva et al., 2021). Recent studies show that the oral administration of BGs could clear the hepatitis B virus in a mouse model (Yu et al., 2015). BGs were also found to activate and enhance cytotoxic T lymphocyte (CTL) responses through the Phosphatidylinositol 3-kinase (PI3K) pathway and were also able to protect against systemic aspergillosis (Kumar and Kumar, 2019). The subcutaneous vaccination of mice with the hepatitis B surface antigen (HBsAg) that was synchronously stimulated with particles containing neutral yeast-derived glucan particles (GPs) showed increased serum IgG. Compared with other kinds of artificial particles, only GPs elicited the secretion of HBsAg-specific Th1, Th2, Th9, Th17, Th22, and Treg-related cytokines (Soares et al., 2019). In addition, early supplementation of *S. cerevisiae boulardii* CNCM I-1079 (SCB) in newborn dairy calves increases IgA production in the intestinal mucosa, and the endogenous IgA production in the gut enhanced *Lactobacillus* sp. formulation and tended to have a broader proportion of *Faecalibacterium prausnitzii* in the jejunum compared with control calves. These results suggest that SCB affects the early development of the immune system and the maturation of the gut microbiome (Villot et al., 2020). This study constructed a new oral yeast agent ST1814G-S (RBD-FP). The result of *in vivo* immunogenicity assay in the mice model reveals that spike antigen-detectable IgG and IgA levels rose after the third round of vaccination (Figure 3). A new COVID study shows that T cell vaccination alone in the absence of neutralizing antibodies could protect mice from SARS-CoV-2 and SARS-CoV infection but not MERS-CoV (Zhuang et al., 2021). Virus-specific memory T cells alone showed the protective role from SARS-CoV-2 disease after Venezuelan equine encephalitis replicon particle (VRP) vaccination (VRPs expressing SARS-CoV-2 structural proteins). With a similar yeast surface display strategy, Lei’s laboratory detected a significant level of neutralizing antibodies against SARS-CoV-2 pseudovirus infection in the yeast EBY100/pYD1-RBD oral administration group (Gao et al., 2021). This result indicated that oral inoculation with recombinant yeast expressing RBD of S-protein effectively blocks spike binding with host receptor and would provide a potential SARS-CoV-2 vaccine candidate against infection. In addition, the RBD of spike protein is an appealing antigen for a subunit vaccine. Another new report provides that yeast-expressed RBD elicits a potent neutralizing antibody response (Chen et al., 2020). This finding lights us design inspiration on antigen selection. The spike residence containing 300–1,000 Amino acid residues, including RBD and FP domain, was selected in our study. The FP was designed and engineered on the surface of the recombinant yeast according to its consistently membrane-perturbing effect. By aligning the spike protein-encoding sequence of SARS-CoV-1, SARS-CoV-2, and MERS, Lai’s laboratory identifies three residues of variability leading to membrane-ordering development and great hydrophobicity. This may be the reason why SARS-CoV-2 appears much more contagious and infectious (Lai and Freed, 2021). Therefore, the recombinant yeast agent with RBD and FP domains would be more efficient than RBD alone. The RBD-FP subunits of spike protein fused encoding Aga2 peptide at the N-terminal expressed in the recombinant yeast (Figure 1). Yeast cells expressing foreign antigens can present through MHC-1 or MHC-2 inducible by APCs, which is recognized by CD81^+^ (cluster of differentiation) CTLs or CD41^+^ T helper cells, respectively, and then leads to proliferation, maturation, and activation of antigen-specific CTLs (Kumar and Kumar, 2019). Our study mainly focuses on the safety evaluation, the roles of gut microflora, and the change in antibody production. The key responsibility of the immune system toward spike RBD-FP involving T cells, γδT, NKT, and ILCs should be further investigated in the future.

The delivery of yeast probiotics after oral administration promotes mucosal immune response. The provoked immunity positively regulates the gut microflora, perhaps, in turn, retaining gut homeostasis by regulating the gut microflora, promoting mucosal immune response, and providing defense against infection. Perturbed mucosal immunity and dysbiosis accompany the clinical disease, including cancers, metabolic diseases, allergies, and immunologic disorders (Fukui et al., 2018). “Long COVID” is the new evidence. The gut microbiome changes are linked to disease severity both in adult patients and young children with COVID-19 (Yeoh et al., 2021; Nashed et al., 2022; Sun et al., 2022). Several gut commensals microbiota with known immunomodulatory potential were underrepresented in patients and remained low in samples collected up to 30 days after disease resolution. Moreover, the gut microbiota composition in COVID-19 patients is concordant with disease severity and magnitude of plasma concentrations of several inflammatory cytokines, chemokines, and blood markers of tissue damage. The pleiotropic effect of probiotics in fighting against influenza virus and other kinds of coronavirus have been estimated, and its role in COVID-19 is awaited from many ongoing clinical trials (Kurian et al., 2021). Tun’s study has identified specific gut microbiota markers associated with improved immune response and reduced adverse events following COVID-19 vaccines (Ng et al., 2022). In our study, the diversity analyses of the gut microbiome reveal that *Moraxella osloensis*, *Roseomonas* sp., and uncultured bacteria *Oscillospira*, *Lachnospiraceae*, and *Nitrospira*, *Burkholderiaceae* were the primary difference between the recombinant yeast and negative control group (*p* < 0.05, Meta Stats). Interestingly, *Lachnospiraceae* and *Burkholderiaceae* were enriched and played immunomodulatory roles in the rumen microbiome on feed efficiency (de Carvalho Filho et al., 2012; Fouhse et al., 2017). Gut microbiota in the ST1814G yeast control group was composed of several species, including *Dickeya chrysanthemi*, *Rhodococcus* sp. B-1016, *Acidisoma tundrae*, *Pseudomonas lurida*, *Methylobacterium extorquens*, *Aeromicrobium erythreum*, and *Megasphaera elsdenii* (*p* < 0.05, Meta Stats) (Supplementary Table 1), but none of them were recorded in immunomodulatory.

Interestingly, in the model of oral administration, recombinant yeast could be detected mainly in female fecal of the high-dose group. This result highlights that yeast agents pass through the stomach, small intestine, and large intestine throughout 4–24 h. Many uncultured bacteria, including *Muribaculaceae*, *Halomonas*, *Ilumatobacter*, *Blastococcus*, *Dietzia*, *Gemmatimonadaceae*, have significant differences between male and female yeast agents’ groups (Supplementary Table 1), which indicated gender differences in the gut microbiota related to diseases, such as irritable bowel syndrome (Mulak et al., 2014). Besides, gender influences both exposure to the virus and susceptibility to severe outcomes. Receptors for sex hormones are also expressed in immune cells, and thus, sex hormones play a role in establishing the sex difference in the immune response (Kim et al., 2020). Takahashi assessed immune phenotype in a sample group of COVID-19 patients and concluded that the number of non-classical monocytes and the level of CCL5 were higher in male patients, and activated CD8 T cell numbers were higher in female patients (Takahashi et al., 2020; Shattuck-Heidorn et al., 2021). Further research raises questions about whether the differences in immune response in men related to gut microbiota diversity, given an explanation of men’s preexisting higher mortality rates before the pandemic.

Overall, our study provides a new oral candidate vaccine against SARS-CoV-2 infection. The recombinant yeast enhanced antigen-specific mucosal and systemic immune responses. The composition of gut microbiota was significantly altered in yeast agent administration compared with the yeast group, perhaps promoting mucosal immune response, retaining gut homeostasis, and providing defense against infection. The study offers a new option with a non-invasive immunization strategy.

## Data Availability Statement

The data presented in the study are deposited in the National Center for Biotechnology Information Sequence Read Archive (NCBI-SRA) repository, accession number PRJNA812734.

## Ethics Statement

The animal study was reviewed and approved by the Institutional Animal Ethical Committee of Tianjin Institute of Pharmaceutical Research [Certificate Number: SYXK (Jin) 2016-0009, Tianjin, China].

## Author Contributions

LZ, LY, YG, XL, LM, JL, RS, and XH performed the laboratory experiments. LM and JL performed the animal experiment analyses with LZ and JH. LZ and XL performed the Gut microbiome composition analyses. YG and JH designed the COVID-19 S gene synthesis. JH contributed to the experimental design. LZ and JH drafted and prepared the manuscript. All authors reviewed the results and approved the final version of the manuscript.

## Conflict of Interest

LM and JL were employed by the Tianjin Institute of Pharmaceutical Research Co., Ltd., China. The remaining authors declare that the research was conducted without any commercial or financial relationships that could be construed as a potential conflict of interest.

## Publisher’s Note

All claims expressed in this article are solely those of the authors and do not necessarily represent those of their affiliated organizations, or those of the publisher, the editors and the reviewers. Any product that may be evaluated in this article, or claim that may be made by its manufacturer, is not guaranteed or endorsed by the publisher.

## Footnotes

1
clinicaltrials.gov

2
www.biocloud.net

3
https://bugbase.cs.umn.edu/
